# Time series covering up to four decades reveals major changes and drivers of marine growth and proportion of repeat spawners in an Atlantic salmon population

**DOI:** 10.1002/ece3.8780

**Published:** 2022-04-02

**Authors:** Alison Harvey, Øystein Skaala, Reidar Borgstrøm, Per Tommy Fjeldheim, Kaja Christine Andersen, Kjell Rong Utne, Ingrid Askeland Johnsen, Peder Fiske, Synne Winterthun, Sofie Knutar, Harald Sægrov, Kurt Urdal, Kevin Alan Glover

**Affiliations:** ^1^ Institute of Marine Research Bergen Norway; ^2^ Faculty of Environmental Sciences and Natural Resource Management Ås Norway; ^3^ 8019 Norwegian Institute for Nature Research Trondheim Norway; ^4^ Rådgivende Biologer Bergen Norway; ^5^ Department of Biology University of Bergen Bergen Norway

**Keywords:** Atlantic salmon, marine growth, salmon lice, sea temperature, veteran spawners, zooplankton

## Abstract

1. Wild Atlantic salmon populations have declined in many regions and are affected by diverse natural and anthropogenic factors. To facilitate management guidelines, precise knowledge of mechanisms driving population changes in demographics and life history traits is needed.
2. Our analyses were conducted on (a) age and growth data from scales of salmon caught by angling in the river Etneelva, Norway, covering smolt year classes from 1980 to 2018, (b) extensive sampling of the whole spawning run in the fish trap from 2013 onwards, and (c) time series of sea surface temperature, zooplankton biomass, and salmon lice infestation intensity.
3. Marine growth during the first year at sea displayed a distinct stepwise decline across the four decades. Simultaneously, the population shifted from predominantly 1SW to 2SW salmon, and the proportion of repeat spawners increased from 3 to 7%. The latter observation is most evident in females and likely due to decreased marine exploitation. Female repeat spawners tended to be less catchable than males by anglers.
4. Depending on the time period analyzed, marine growth rate during the first year at sea was both positively and negatively associated with sea surface temperature. Zooplankton biomass was positively associated with growth, while salmon lice infestation intensity was negatively associated with growth.
5. Collectively, these results are likely to be linked with both changes in oceanic conditions and harvest regimes. Our conflicting results regarding the influence of sea surface temperature on marine growth are likely to be caused by long‐term increases in temperature, which may have triggered (or coincided with) ecosystem shifts creating generally poorer growth conditions over time, but within shorter datasets warmer years gave generally higher growth. We encourage management authorities to expand the use of permanently monitored reference rivers with complete trapping facilities, like the river Etneelva, generating valuable long‐term data for future analyses.

## Graphical abstract

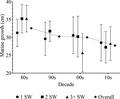

## 1 INTRODUCTION

Wild Atlantic salmon (*Salmo salar*) face a complex suite of environmental stressors throughout their lives. Some of these stressors are natural, while others are caused by constantly expanding anthropogenic activities in rivers and the coastal zone (Forseth et al., 2017; Lennox et al., 2021). With some exceptions in the northern areas (Niemelä et al., 2005), Atlantic salmon (hereon referred to as salmon) populations have declined throughout most of their distribution over the past several decades (Friedland et al., 2009; Jensen et al., 2011; Peyronnet et al., 2007, 2008; Todd et al., 2008). Parasites like salmon lice *Lepeophtheirus salmonis* (Thorstad et al., 2015) and *Gyrodactylus salaris* (Johnsen & Jensen, 1991), introgression of escaped domesticated salmon (Bolstad et al., 2017; Fleming et al., 2000; Glover et al., 2013, 2019; McGinnity et al., 2003; Skaala et al., 2019), river regulations and agriculture practices have all been identified as major threats to the abundance of salmon populations, although their relative importance varies from region to region and over time (Forseth et al., 2017).

It is also becoming increasingly evident that climate change, by influencing physical and biological conditions in both the freshwater and marine phase of the salmon´s anadromous life cycle, is likely to directly and indirectly influence survival, production, and distribution of wild salmon populations (Beaugrand & Reid, 2003; Friedland et al., 2009; Jensen et al., 2011; Tréhin et al., 2021). It is, therefore, necessary to investigate a diverse range of factors, from direct anthropogenic to climatic, in order to identify and quantify the mechanisms underpinning variation in growth and population abundance in salmon (Chaput, 2012; ICES, 2013; NASCO, 2002, 2009). In order to elucidate some of these processes, earlier studies have investigated, with contrasting results, correlations between angling catch reports, marine return rates or post‐smolt growth, and climate variables such as sea surface temperatures (SST) and the North Atlantic Oscillation (NAO) index, and the biomass of pelagic fish species (Bacon et al., 2009; Beaugrand & Reid, 2003; Friedland et al., 2000; Jensen et al., 2012; Quinn et al., 2006; Todd et al., 2008; Utne et al., 2020). Other studies (Brett, 1979; Friedland et al., 2000, and references therein) found marine growth rate, particularly during the post‐smolt period, to be correlated with sea temperature and prey abundance. As marine growth rate and survival are partially linked (Friedland et al., 2000; Jonsson et al., 2003), environmental factors affecting marine growth rate, caused by either human activities or natural variations, represent key elements in our understanding of variations in population abundance, and ultimately, how to manage these populations.

The overall aim of the present study was to investigate temporal variation in marine growth rate of salmon during their first year at sea, age at maturation, the proportion of repeat spawners in the population, and finally, to identify potential drivers of variation in marine growth. These analyses were conducted on a unique dataset from the river Etneelva using the following three sources of data: (a) angling reports and scale samples covering four decades, (b) extensive sampling of the whole spawning run from 2013 onwards in an upstream migration trap, and finally (c) an environmental time series of sea surface temperature, zooplankton biomass, and sea lice intensity spanning up to four decades.

## 2 MATERIALS AND METHODS

### 2.1 Study design

The study consisted of two datasets: (1) salmon captured during the angling season (mid‐June to mid‐August) for intermittent years 1983 to 2019, with date of capture and biological measurements for each fish, (2) salmon captured in the upstream migration trap, with date of capture and biological measurements for each individual fish entering from April to November (2013 to 2019). The angling data were collected by the Institute of Marine Research (IMR) and the Norwegian Institute for Nature Research (NINA; Table 1). In addition, measurements of sea surface temperature, biomass of zooplankton, and median salmon lice intensity were also compiled from various sources for the various years in the study period (Supplementary data for more information). Average marine growth for the trap and angling datasets are presented (Table S1).

**TABLE 1:** Number of wild salmon for each sampling method (trap and angling) used in the analyses pertaining to this study from the river Etneelva from 1983 to 2019. The total number of salmon caught by angling and ascending the trap are shown for each year, and the number of salmon divided into sexes, spawning status, and sea ages for each year are also shown. The source of the angling samples is shown in brackets; IMR: Institute of Marine Research, NINA: Norwegian Institute for Nature Research

A Sample source	Year	Total *N*	Sex		Spawning status		Sea age			Marine growth
			Female *N*	Male *N*	Maiden *N*	Repeat *N*	1 year *N*	2‐year *N*	3+ year *N*	*N*
Angling (IMR)	1983	472	88	88	479	10	361	49	61	466
Angling (IMR)	1984	547	165	246	578	16	315	175	57	545
Angling (NINA)	1989	123	30	42			89	28	6	123
Angling (NINA)	1990	1	1				1			
Angling (NINA)	1992	17	8	8			13	4		17
Angling (NINA)	1994	19	7	11			16	3		19
Angling (NINA)	1997	14	4	8			14			14
Angling (NINA)	1998	22	9	9			7	15		22
Angling (NINA)	2000	26	11	14			14	12		26
Angling (NINA)	2002	23	10	10			17	6		23
Angling (NINA)	2004	21	11	9			6	15		21
Angling (NINA)	2005	22	9	10			17	5		22
Angling (NINA)	2006	39	17	20			16	22	1	39
Angling (NINA)	2007	22	12	9			11	11		22
Angling (NINA)	2008	52	22	22				37	15	52
Angling (NINA)	2010	9	8	1				9		9
Angling (NINA)	2011	11	7	4			1	9	1	11
Angling (NINA)	2012	185	66	95			23	98	62	185
Angling (IMR)	2013	182	63	94	172	34	29	70	83	182
Angling (IMR)	2016	335	129	179	346	21	30	260	45	335
Angling (IMR)	2017	299	126	158	279	46	37	146	115	299
Angling (IMR)	2018	96	48	47	105	9	22	60	13	96
Angling (IMR)	2019	171	58	100	168	16	45	89	36	171
Trap	2013	1141	635	506	1041	100	265	494	315	116
Trap	2014	411	179	232	336	75	148	133	118	393
Trap	2015	2152	742	1410	2143	9	1128	767	133	227
Trap	2016	2164	1241	923	2153	11	365	1527	145	213
Trap	2017	1900	961	937	1672	228	488	880	485	1835
Trap	2018	1538	766	772	1396	142	501	782	215	1494
Trap	2019	1210	498	712	1125	85	466	503	224	1163

### 2.2 The river Etneelva

**FIGURE 1:**
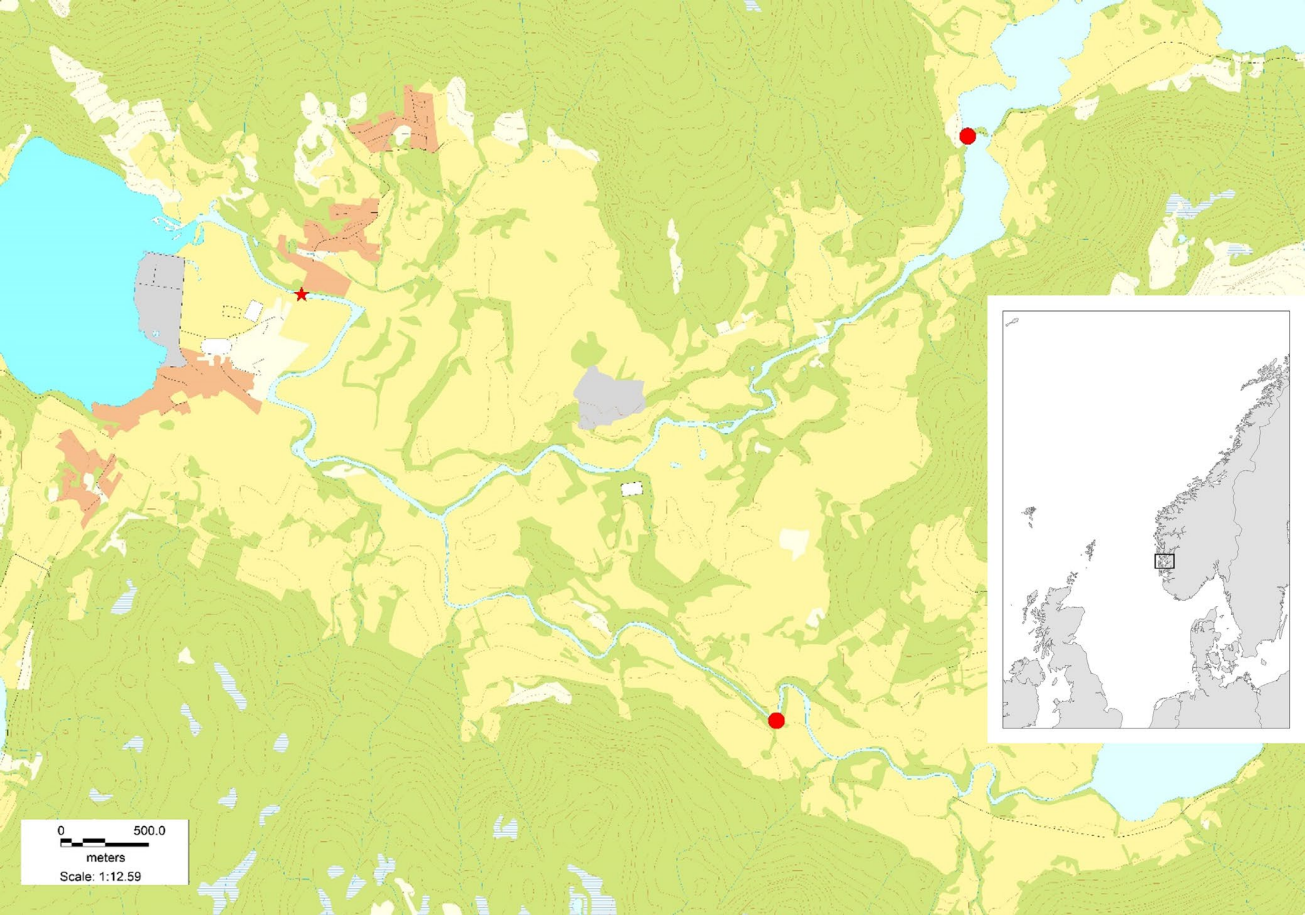
Map of the river Etneelva showing the location of the upstream migration trap (red star) and location of two measuring stations for river water discharge and river water temperature (red circles)

The river Etneelva is located near the mouth of the Hardangerfjord on the west coast of Norway (Figure 1). The anadromous section is ~13 km, covering ~290 000 m^2^ of habitat. In 2013, a resistance board weir fish trap was installed in the lower part of the river to monitor and sample the spawning runs for salmon and anadromous brown trout (*Salmo trutta*) (Harvey et al., 2017; Madhun et al., 2017; Quintela et al., 2016; Skaala et al., 2015). The trap is also used to remove putative escaped domesticated salmon (Madhun et al., 2017). For each fish that enters the trap, the species (salmon or trout), sex, length, and weight were recorded. A small number of scales were taken from each fish for age and growth analyses (sampled above the lateral line between the dorsal and adipose fin), and a micro‐clip was taken from the tip of the adipose fin for genetics, before wild fish were released above the trap. Based on sub‐sampling methods and snorkeling counts, the catch efficiency of the trap has been estimated at approximately 98% for escaped domesticated salmon and slightly less for wild salmon (Skoglund et al., 2021).

The study was conducted in agreement with the Vestland County Governor, the Norwegian Environmental Agency and the Norwegian Food Safety Authority with permits (No. 2015/34273‐1 and No. 19/36679/‐1) to capture, sample, and tag salmon.

### 2.3 Age and growth analyses

For determination of age and growth, rinsed scales were photographed with calibration using a stereomicroscope. The number of years in freshwater until smoltification, the number of winters in the sea, and the occurrence of spawning marks were determined. For salmon captured in the trap in 2015 and 2016, only a random subset (every tenth fish entering the trap) of individuals’ scales were analyzed; therefore, the number of repeat spawners in those years are lower (and not representative) than in other years where all fish scales were analyzed (see Table 1). In addition, smolt length was back‐calculated for a subset of individuals captured in the trap and all angled individuals using the methodology described by Lea‐Dahl (Dahl, 1910; Lea, 1910; Table S1).

### 2.4 Statistics

#### 2.4.1 Marine growth during the first year at sea

All statistics were carried out using R v4.1.2 (R Core Team, 2016). Generalized linear models were used to investigate variations in marine growth during the first year of the fish caught by angling and a subset of the fish caught in the trap. The response variable was marine growth, measured as the post‐smolt growth increment (PGI), and calculated by subtracting the back‐calculated smolt length from the estimated length after the first winter at sea. Marine growth was modeled using a Gaussian distribution with a log‐link function using *glmmTMB* function from the glmmTMB package in R (Brooks et al., 2017) in all models unless stated otherwise. As certain variables of interest were present in different subsets of years, it was decided to investigate marine growth using different models, depending on the availability of the data. The analyses were, therefore, split into demographic and environmental models for each dataset, that is, two models for the angling dataset and two models for the trap dataset. In the demographic model for the angling data, smolt year classes (ranging from 1980 to 2018) were grouped into decades, modeled as an explanatory variable consisting of four levels (80s, 90s, 00s, and 10s). The other explanatory variables included in the model were the sex (two levels: male or female), and sea age (three levels: 1, 2 or multi‐sea winter (MSW)) of each fish, with a two‐way interaction for sex and decade. All variables were modeled as categorical variables, and decade was included in the dispersion formula to account for heteroscedasticity. The demographic model for the trap data included the explanatory variables of sex (two levels: male or female), sea age (three levels: 1, 2, or MSW years), and the smolt year class of the fish (years containing complete smolt year classes, 5 levels: 2012–2016). The interactions between sex and smolt year class and between sea age and smolt year class were included as two‐way interactions. All variables were modeled as categorical variables and smolt year class was included in the dispersion formula. For the angling data, there were two environmental variables of interest, average summer sea surface temperature (SST) and average May zooplankton biomass; however, the coverage of these variables over the study period differed. Data pertaining to SST were available for the entire angling dataset period (intermittent smolt year classes 1980–2018), while zooplankton data were only available for smolt year classes from 1996 to 2018. Therefore, it was decided to investigate the influence of SST and zooplankton on marine growth in one model relating to the shorter time (1996–2018) where SST and zooplankton were both modeled using smooth functions and decade was included as a random smooth term. A model relating to the entire study period (smolt year classes 1980–2018) was also fitted with SST modeled as above and smolt year class modeled as a random smooth term. These two environmental models were fitted using the *gam* function from the mgcv package in R (Wood, 2017) with a Gaussian distribution with a log‐link function. For the trap data, the explanatory variables for the environmental model were the estimated average salmon lice intensity for the river Etneelva, the average zooplankton index for May, and the average summer sea surface temperature (SST), all relating to individual smolt year (here, smolt year classes 2012–2018 were included) and modeled as continuous variables. Smolt year class was included in the dispersion formula as above, and the model was fitted using a Gaussian distribution with a log‐link function using *glmmTMB* as above.

Model fits were assessed by using the DHARMa package in R (Hartig, 2022). The *Anova* function from the car package (Fox & Weisberg, 2019) was used to assess the significance of the explanatory variables for the glm models, and *anova.gam* was used to assess the significance of the smooth terms for the gam models. For the significant main categorical variables with more than three levels and for significant two‐way interactions, pairwise comparisons between each level of the factor were carried out using the *pairs* function from the emmeans (estimated marginal means) package (Lenth, 2016) with the default Tukey adjustment for multiple comparisons.

#### 2.4.2 Age at maturation

A series of two‐proportion Z tests were used to investigate the difference in proportions of salmon of each sea age between the decades of angling and between the years of capture in the trap to explore potential shifts in age at maturation over time. *p* values were adjusted for multiple comparisons using a Bonferroni correction.

#### 2.4.3 Sea residency of repeat spawners

Two‐proportion Z tests were used to assess differences in the proportion of repeat spawners observed in historical (1983 + 1984) and contemporary (2018 + 2019) angling samples, between sexes within the trap and angling samples. 2018 and 2019 were used as these represented the most contemporary samples that contained complete estimation of repeat spawners.

## 3 RESULTS

### 3.1 Marine growth during the first year of the salmon captured by angling

**FIGURE 2:**
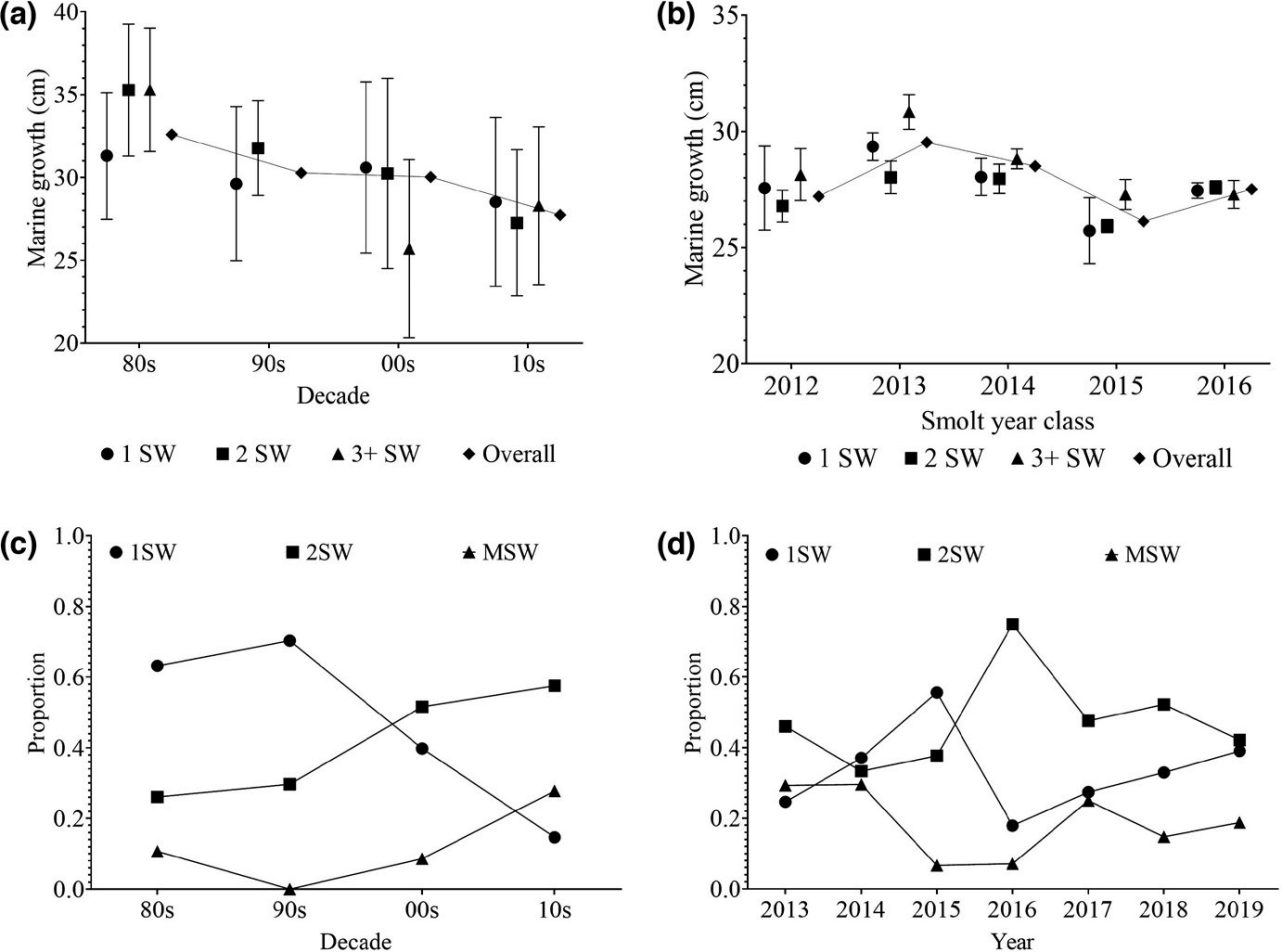
Marine growth during the first sea winter of (a) angling fish of different sea ages captured by the river trap for each decade and (b) fish of different sea ages captured by the trap for each smolt year class. Marine growth is represented by the average and 5–95% confidence intervals. Proportions of fish of each of sea age for (c) each decade of capture for the angling fish and (d) each year of capture for the trap fish

Marine growth to the first annual zone, that is, at the completion of the first summer and winter at sea, was significantly associated with decade and sea age, while neither sex nor its two‐way interaction with decade was significantly associated with marine growth (Table 2A: Figure 2a). Averaged over all the years, 1 SW fish were significantly larger than both 2 SW and MSW fish (*t* ratio = −7.12, *df* =2041, *p* value = < .000 and *t* ratio = −6.93, *df* = 2041, *p* value = < .000, respectively), while 2 SW were smallest, although average size differences were very small (1SW: 30.41 cm, 2SW 29.01 cm, MSW: 29.24 cm) and there were no clear trends among the decades Marine growth displayed a distinct decline over time, with the lowest average marine growth observed in the 10s (Figure 2a). Post hoc pairwise comparisons between decades revealed that marine growth was significantly different between all decades, with fish caught in the 1980s being on average 5 cm larger than fish caught in the 2010s (Table S2).

**TABLE 2:** Anova output of the generalized linear models and generalized additive models investigating the factors influencing the marine growth of Atlantic salmon from the river Etneelva after the first winter at sea for fish captured by angling (A—demographic model and B—environmental model) and in the trap (C—demographic model and D—environmental model)

A	Model terms	Chi‐square	*df*	*p* value
A	Sex	1.27	1	.259
	**Sea age**	**49.64**	**2**	**<.000**
	**Decade**	**591.14**	**3**	**<.000**
	Sex × Decade	1.05	3	.790

B		edf	*F*	*p* value
	**s(Zooplankton)**	**3.81**	**5.59**	**.000**
	**s(Summer SST)**	**3.93**	**9.86**	**<.000**
	**re(Decade)**	**1.74**	**5.24**	**.003**

C		Chi‐square	*df*	*p* value
	**Sex**	**12.07**	**1**	**.001**
	**Smolt year**	**181.66**	**4**	**<.000**
	**Sea age**	**35.08**	**2**	**<.000**
	Sex × Smolt year	1.18	4	.882
	**Sea age** × **Smolt year**	**27.91**	**8**	**.000**

D		Chi‐square	*df*	*p* value
	**Sea lice**	**35.66**	**1**	**<.000**
	**Zooplankton**	**17.92**	**1**	**<.000**
	**Summer SST**	**199.26**	**1**	**<.000**

Note

Significant terms are shown in bold. *df*, degrees of freedom.

**FIGURE 3:**
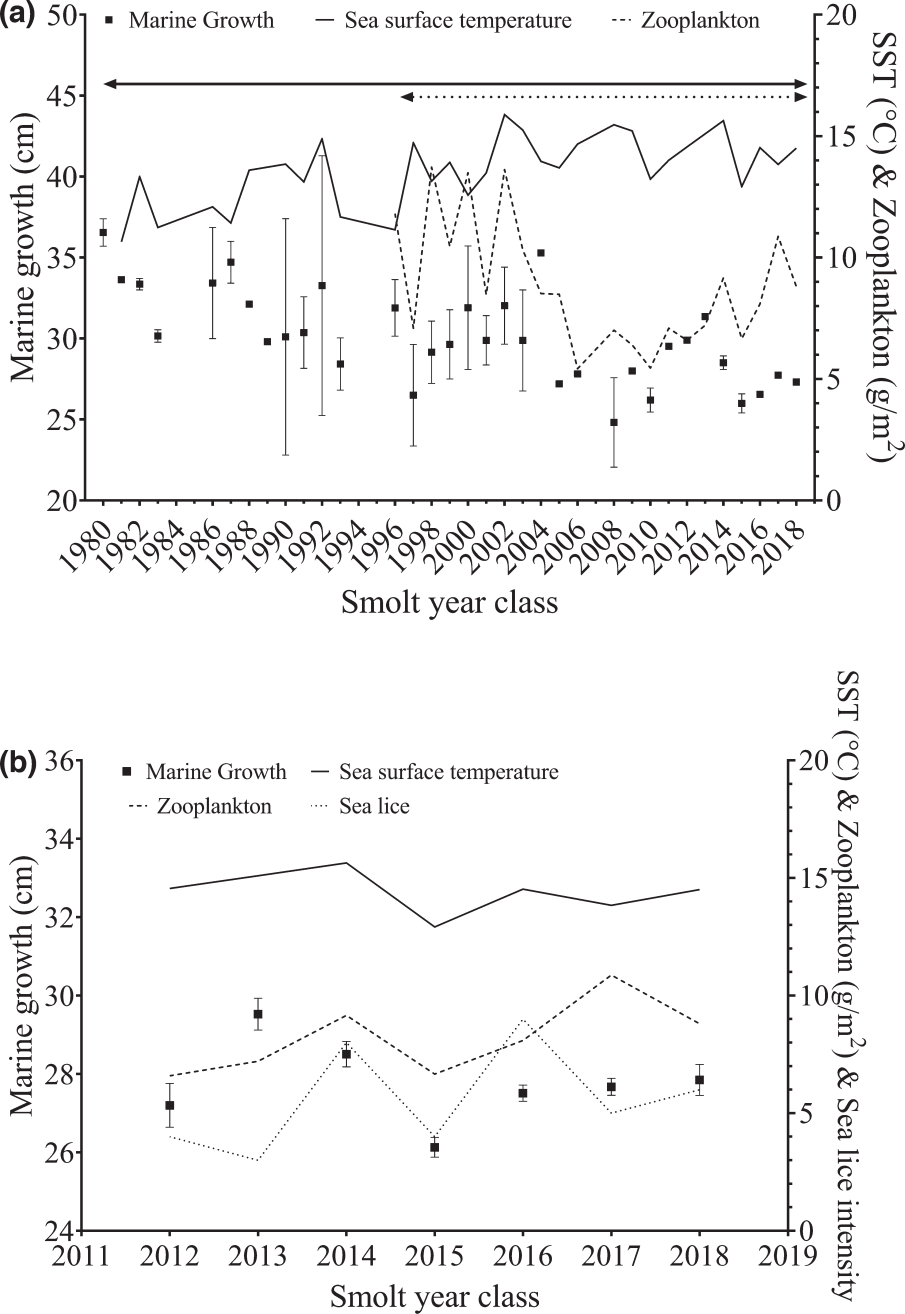
Marine growth to first annual zone of (a) salmon captured by angling in the period 1983–2018 and (b) salmon captured in the trap in 2013–2019, with their corresponding years of smoltification and exiting the river. Marine growth is represented by the average and 5–95% confidence intervals. Seasonal summer sea surface temperature (SST) (°C) (solid line), average May biomass of meso‐zooplankton (g/m^2^) (dashed line), and median intensity of salmon lice (stippled line) are also shown for each smolt year class. The horizontal line and stippled line above the data in window A represent the two time periods analyzed in the two environmental models relating to the angling data

In the environmental models, the results for the relationship between marine growth during the first year at sea and average summer sea surface temperature differed depending on the model (i.e., depending on the length of the time series used) and, therefore, needs to be carefully interpreted. With the model containing both the summer SST and biomass of zooplankton (consisting of smolt year classes 1996–2018), both smooth terms were nonlinear and significant (Table 2b). Marine growth significantly increased with zooplankton biomass values and SST in a nonlinear manner (Figure 3a, Table 2d). In the model containing only summer SST as a covariate (here, the entire study period of smolt year classes 1980–2018), the relationship between marine growth and the average summer SST was linear, significant, and negative (*F* value = 6.06, estimated *df* = 1, *p* value = .012; Figure 3a).

### 3.2 Marine growth during the first year of salmon captured in the trap

Marine growth was significantly different between the smolt year classes (2012–2016), sea ages, and sexes (Table 2c). On average, females were significantly smaller than males, although the difference was small (27.25 versus 27.72 cm). The interaction between sea age and smolt year class was significant (Table 2c). For smolt year classes 2012–2015, MSW displayed significantly larger marine growth compared with 2 SW fish (Table S3, Figure 2C), and in 2013 1 SW fish also had significantly higher growth than 2 SW fish (Table 3). Although the differences were not always significant, MSW fish were the largest in all smolt year classes apart from 2016, while 2 SW fish were the smallest in smolt year classes 2012–2014, intermediate in 2015 and had the largest marine growth in 2016 (Figure 2c).

**TABLE 3:** Bonferroni‐adjusted *p* values for the multiple two‐proportion Z test comparisons between the proportions of fish within each sea ages within (A) the decades of capture by angling and (B) the years caught in the trap

A	90s	00s	10s
1SW			
80s	1.000	.000	.000
90s		.001	.000
00s			.000
2SW			
80s	1.000	.000	.000
90s		.070	.000
00s			1.000
MSW			
80s	.290	1.000	.000
90s		1.000	.000
00s			.000

B	2014	2015	2016	2017	2018	2019
1SW						
2013	.000	.000	.001	1.000	.000	.000
2014		.000	.000	.001	1.000	1.000
2015			.000	.000	.000	.000
2016				.000	.000	.000
2017					.000	.000
2018						.199
2SW						
2013	.001	.000	.000	1.000	.083	1.000
2014		1.000	.000	.000	.000	.080
2015			.000	.000	.000	.494
2016				.000	.000	.000
2017					.283	.160
2018						.000
MSW						
2013	1.000	.000	.000	1.000	.000	.000
2014		.000	.000	1.000	.000	.000
2015			1.000	.000	.000	.000
2016				.000	.000	.000
2017					.000	.000
2018						.085

Marine growth was significantly associated with all environmental covariates (Figure 3b; Table 2D). Over the shorter study period of the trap data (smolt year classes 2012–2018), summer SST was positively associated with marine growth, with individuals from smolt year classes with higher average summer sea surface temperatures displaying higher marine growth. Similarly, for smolt year classes in years with high May biomass of zooplankton, marine growth was significantly higher than individuals migrating out in years with lower biomass of zooplankton. Marine growth was negatively associated with the median intensity of salmon lice, with fish originating from smolt year classes with high salmon lice intensity displaying a lower average marine growth during the first year in the sea (Table 2D; Figure 3b).

### 3.3 Age at maturation

The proportion of 1SW salmon caught by angling decreased significantly between the 1980s and 2010s. The proportion of 1SW was lower in the 80s than in the 90s albeit this difference was not significant. The proportion of 1SW was significantly lower in the 2010s than in every other decade, dropping from 0.70 in the 90s to 0.15 (Table 3; Figure 2c). The opposite trend was observed in 2SW fish, with significantly higher proportions of 2SW fish caught by angling in the 2000s and 2010s than in the previous two decades (Table 3; Figure 2c). The proportion of MSW salmon caught by angling was significantly higher in the 2010s compared with every other decade, while there were no differences in proportions observed between the 80s, 90s, and 2000s (Table 3; Figure 2c).

The proportion of 1SW fish caught in the trap varied over the years without a clear trend (Table 3; Figure 2d). The proportion of 1SW increased significantly from 2013 to a high in 2015; however, there was then a significant decrease in 1SW in 2016, where the proportion of 1SW fish was significantly lower than all other years. After 2016, the proportion of 1SW fish increased again. Similarly, there was no clear trend in the change in proportion of the 2SW fish caught in the trap over the years (Table 3; Figure 2d), although in 2016 the proportion of 2SW fish was significantly higher than all other years. The proportion of MSW was highest in 2013 and 2014 and lowest in 2015 and 2016; however, statistical significance of the proportional differences varied among the years and there was no clear trend (Table 3; Figure 2d).

### 3.4 Proportion of repeat spawners

The total proportion of repeat spawners in the population was significantly lower in the historical (1983 & 1984) angling samples (3%) compared with the contemporary (2018 & 2019) angling samples (7%) (Table 4A). Within sexes, there were significantly lower proportions of female repeat spawners in the historical angling samples (2%) compared with the contemporary angling samples (9%) (Table 4A). This trend was also evident for the males, but statistically not significant (historical: 3% and contemporary: 6%) (Table 4A).

**TABLE 4:** Two‐proportion z tests comparing the proportion of repeat and maiden spawners between (A) historical vs contemporary samples and (B) trap and angling samples of Atlantic salmon from the river Etneelva

A	Comparisons	Historical (1983 +1984)	Total (*n*)	Proportion	Contemporary (2018 +2019)	Total (*n*)	Proportion	Chi‐square	*df*	*p* value
	**Angling P**	**18**	**612**	**0.03**	**20**	**276**	**0.07**	**7.588**	**1**	**.006**
	**Angling F**	**6**	**263**	**0.02**	**11**	**117**	**0.09**	**8.013**	**1**	**.005**
	Angling M	12	349	0.03	9	159	0.06	0.858	1	.354

B	Comparisons	Trap	Total (*n*)	Proportion	Angling	Total (*n*)	Proportion	Chi‐square	*df*	*p* value
	**All years P**	**630**	**6198**	**0.1**	**132**	**1703**	**0.08**	**8.6555**	**1**	**.003**
	**All years F**	**435**	**3039**	**0.14**	**56**	**716**	**0.08**	**20.294**	**1**	**<.001**
	All years M	195	3159	0.06	76	987	0.08	2.6268	1	.105

Abbreviations: *df*, degrees of freedom; F, females; M, males; P, pooled sexes; RSP, repeat spawners.

Significant terms are shown in bold.

The total proportion of repeat spawners in the contemporary samples (2018 + 2019) was significantly lower in those captured by angling (8%) compared to those ascending the trap in the same years (10%) (Table 4B). There were significantly less females in the angling (8%) than in the trap samples (14%), but no difference between proportions of males in the angling (8%) and the trap samples (6%) (Table 4B). The proportion of repeat spawners fluctuated among the years, although in 2014, a year with low salmon returns, the relative number of repeat spawners was high (Table 1), with female repeat spawners constituting 43% of the female biomass and fecundity.

## 4 DISCUSSION

Using a dataset spanning smolt year classes 1980–2018, we observed a clear temporal decline in growth rate during the first year at sea, with a stepwise reduction over the four decades. In the same time period, we also observed a clear switch from a dominance of 1SW fish to a dominance of 2SW fish and more than a doubling in the proportion of repeat spawners in the population. The influence of summer SST on marine growth depended on the length of the time series used, with a negative effect over the longer angling time series, and a positive effect over the shorter fish‐trap time series. Zooplankton positively influenced marine growth, while sea lice intensity negatively influenced growth. This is the first study to investigate the combined influence of SST, zooplankton biomass, and sea lice intensity on marine growth in salmon. We conclude that both changing oceanic conditions over time and anthropogenic activities have contributed to these clear changes in the population demography and age structure.

### 4.1 Marine growth rate and age at maturation

A very clear decline in marine growth in the first year at sea was observed over the smolt year classes from 1980 to 2018. A similar temporal reduction in marine growth has also been reported in several other long‐term studies of Atlantic salmon populations in the Northeast Atlantic (Bacon et al., 2009; Fiske et al., 2008; Peyronnet et al., 2007; Smith et al., 2007; Todd et al., 2008).

The observed temporal reduction in growth rate for fish of all age groups during the first year at sea was accompanied by a temporal shift in the proportion of sea age groups in favor of 2SW and MSW fish. Jonsson et al. (2016) found a similar decrease in size and proportion of 1SW of Atlantic salmon in the River Imsa in Norway over the period 1976–2010. Similarly, Otero et al. (2012) studied angling catches in 59 Norwegian rivers over a 15‐year period and reported an overall increase in the age at maturity from 1SW to 2SW fish.

In the present study, marine growth in the first year at sea was statistically associated with the subsequent age at maturation; however, the direction of the response varied from year to year and between periods. For example, in some years and periods the fastest growing fish up to the first winter at sea entered a MSW strategy, while in other years the fastest growing fish entered the 1SW strategy. Therefore, our data are inconclusive regarding this issue. Previous studies have also investigated this phenomena, reporting better growth during the first year at sea in MSW salmon in 7 populations along the Norwegian coast by Jensen et al. (2011), and by Sægrov et al. (2004) who in addition reported a temporal reduction in differences in growth rate among sea age groups in smolt year classes 1975–2002 from the river Suldalslågen, just south of the river Etneelva.

### 4.2 Environmental drivers of marine growth

Growth rate in fish is closely linked with temperature, and with increasing sea temperatures during the last decades, it could be expected that marine growth of Atlantic salmon would increase with time. Our analyses of the effect of sea surface temperature on marine growth covered differing time scales with divergent results. The full angling dataset covering smolt year classes 1980–2018 revealed a negative effect of average summer sea surface temperature on marine growth, while in the shorter angling dataset covering smolt year classes 1996–2018 the effects were nonlinear but positive overall. Likewise, in the trap dataset covering smolt year classes 2012–2018 the effect of SST on marine growth was positive. Earlier studies have also found conflicting influences of SST on marine growth (Bacon et al., 2009; Jensen et al., 2011; Todd et al., 2020), highlighting the fact that conclusions concerning drivers of marine growth rates of Atlantic salmon may differ among studies covering different regions and time periods. Long‐term studies by Todd et al. (2020) and Jonsson et al. (2016) also observed a negative effect of SST on marine growth in Atlantic salmon populations. It has been postulated that increasing SST causes an indirect negative effect on growth through climate changes influencing prey availability (Jonsson et al., 2016; Todd et al., 2008, 2020). In the present study, marine growth fell to an all‐time low for the smolt year classes around 2007, just as zooplankton abundance dropped sharply from a high level at about 10–15 g/m^2^ down to about half the biomass (Figure 3A). A drop in marine growth being correlated with a decrease in zooplankton availability has also been observed by others (Beaugrand & Reid, 2003; Friedland et al., 2009; Todd et al., 2008). Jensen et al. (2012) identified associations between biomass of pelagic fishes (SSB), zooplankton biomass, and growth rate in salmon.

In the trap dataset, we also observed a significant and negative effect of sea lice intensity on marine growth. The potential negative effects from salmon lice on marine growth and survival of anadromous salmonid species have been debated for several decades, particularly in relation to areas with high density of salmon farming (Grimnes & Jakobsen, 1996; Krkosek et al., 2007; Shephard & Gargan, 2021; Skilbrei & Wennevik, 2006; Vollset et al., 2018). Although our data did not allow for a full study on the impact from salmon lice on the survival of salmon, we have expanded existing knowledge on drivers, including salmon lice, of marine growth in a naturally recruited salmon population.

### 4.3 The proportion of repeat spawners

The striking increase observed in the proportion of repeat spawners in the population through the period from 1980 to 2018 is most likely caused by a reduction in mortality of fish following their first spawning event. This could occur in the river or the sea, or a combination. By 1984, Norwegian salmon were heavily exploited upon their migratory return to the coastline, with 21 210 drift nets, 1 697 bag nets, and 35 lift nets in operation in the Norwegian home water fishery (Hansen, 1988). The marine exploitation rate of smolt year classes between 1981 and 1984 from the river Imsa in southwestern Norway was estimated at >90% for 2 SW salmon but somewhat lower for 1 SW salmon. With such a high exploitation rate, it could be expected that fewer fish survive for a second spawning migration. Following the strong regulations on sea fisheries for salmon, introduced by the Norwegian Government in 1986, including a total ban of drift net fisheries (Hansen, 1988), in combination with a relatively low estimated angling mortality in the river Etneelva compared with other studies (Borgstrøm et al., 2010; Erkinaro et al., 1999; Hansen, 1990), an increase in the proportion of repeat spawners in wild salmon populations in this area, and especially the river Etneelva, would be expected. Similar increases in repeat spawners have been observed in Canada due in part to size restrictions on the recreational fishery (Reid & Chaput, 2012). Erkinaro et al. (2019) examined four decades of scale samples from salmon fisheries in the Teno River in northern Europe. The authors found an increase in repeat spawners over time, which they attribute to changes in both fishery exploitation and environmental conditions. Repeat spawners are of particular importance in years with low maiden return, for example, in 2014 where low returns of salmon were observed but a high proportion of repeat spawners relative to other years, and the drivers behind observed spatio‐temporal changes have been addressed by a number of studies (Bordeleau et al., 2020; Hansen, 1988; Peyronnet et al., 2007).

Most of the repeat spawners identified in this study returned as alternate spawners, that is, two years after the previous spawning, as opposed to consecutive spawners the year after. However, this differed between the sexes, as males more often than females tended to return as consecutive spawners. The positive association between female size and fecundity, egg size and energy content (Bordleau et al., 2020; Fleming, 1996), may suggest that egg quality is affected by reconditioning strategy (Reid & Chaput, 2012). In turn, this may explain why an alternative strategy was more commonly observed in females than in males.

The underrepresentation of female repeat spawners relative to males in the angling catches compared with their overrepresentation in the trap suggests intersexual differences in behaviors and therefore angling catchability. This would be in accordance with behavioral differences observed between males and females during the spawning season in salmon (Fleming, 1996) and in anadromous brown trout (Johnsson et al., 2001), where males spend relatively more energy in aggressive contests with other males cruising up and down the river, looking for spawning opportunities, while females use energy in selecting and defending spawning sites.

### 4.4 Management Implications

Our study revealed that changes in marine growth in the first year at sea and in the age and spawning structure of the population have occurred due to changes in oceanic conditions and anthropogenic activities. Determining such changes and their drivers and elucidating how these processes and activities influence salmon populations is key to mitigating and predicting future population changes. Time series, like those used in the present study, and infrastructure with resources like the trapping facility on the river Etneelva are scarce. Still, they are fundamental tools for studying and analyzing changes in population demography over time and among regions and are vital for the sustainable management of wild salmon populations.

## CONFLICT OF INTEREST

None declared.

## AUTHOR CONTRIBUTION

**Alison Harvey:** Formal analysis (lead); Methodology (equal); Software (equal); Writing – original draft (equal). **Øystein Skaala:** Conceptualization (lead); Data curation (equal); Formal analysis (supporting); Funding acquisition (equal); Investigation (lead); Methodology (equal); Project administration (lead); Resources (equal); Software (supporting); Supervision (equal); Validation (equal); Visualization (equal); Writing – original draft (equal); Writing – review & editing (equal). **Reidar Borgstrøm:** Conceptualization (equal); Writing – original draft (equal). **Per Tommy Fjeldheim:** Data curation (equal); Investigation (equal); Methodology (equal). **Kaja Christine Andersen:** Data curation (equal); Investigation (equal). **kjell utne:** Conceptualization (equal); Data curation (equal); Methodology (equal). **Ingrid Johnsen:** Conceptualization (equal); Data curation (equal); Formal analysis (equal); Investigation (equal); Writing – original draft (supporting). **Peder Fiske:** Conceptualization (equal); Data curation (equal); Methodology (equal). **Synne Winterthun:** Data curation (equal); Investigation (equal). **Sofie Knutar:** Data curation (equal). **Harald Sægrov:** Conceptualization (equal); Data curation (equal); Methodology (equal). **Kurt Urdal:** Conceptualization (equal); Data curation (equal); Methodology (equal). **Kevin Alan Glover:** Conceptualization (equal); Data curation (equal); Methodology (equal); Writing – original draft (equal).

## Supporting information

Table S1‐S3

## Data Availability

The raw data underlying the study consist of 8188 individual salmon spawners. These, and the metadata, will be archived and made accessible at the storage facilities at the Norwegian Marine Data Center (NMDC) at the Institute of Marine Research, Bergen, Norway. The data are also available in the Dryad repository: https://doi.org/10.5061/dryad.59zw3r29m.
