# Supplementary material for: Time series covering up to four decades reveals major changes and drivers of marine growth and proportion of repeat spawners in an Atlantic salmon population
Source: Ecol Evol. 2022 Apr 2;12(4):e8780. doi: 10.1002/ece3.8780 (PMC8976282; doi:10.1002/ece3.8780)
Supplement: Supplementary file 1 — Table S1‐S3 [file ECE3-12-e8780-s001.docx]

**Appendix 1: Tables & Figures**

**Additional environmental data**

Sea surface temperature at 1 m depth (°C) (SST) was measured twice a month at Utsira (59°31’N, 4°78’E; <http://www.imr.no/forskning/forskningsdata/stasjoner>). The SST data was obtained for the study period, with a gap in the data from August 2011 – February 2014. Average summer SST for the months of June-August were used in the analysis. Data pertaining to average yearly biomass of meso-zooplankton (g dry weight/m^2^ , sampled in May) in the uppermost 200m of the Norwegian Sea was obtained for the period 1995 – 2018 (ICES, 2020). Finally, yearly estimates of the median salmon lice intensity for the population of salmon from the river Etneelva were obtained from Johnsen et al. (2020) for the period 2012 – 2019. These estimates are based on a model which calculates the estimated mortality due to salmon lice infection pressure during a 4-week smolt migration window (24 April – 3 June for the river Etneelva (Johnsen et al., 2020)). The data pertaining to these environmental parameters can be found in the attached raw data file, apart from the zooplankton data which is available on request.

Table S1: Average marine growth in the first sea winter for the (A) angling-caught fish and the (B) trap-caught fish based on smolt year class. Marine growth is presented as an average ± 1 standard deviation for all smolt year classes and spilt into demographic categories (sex and sea age). Number of fish per sea age is given in brackets.

|  |  |  | Sex | | Sea age | | |
| --- | --- | --- | --- | --- | --- | --- | --- |
| Sample source | Year | Marine Growth  (cm ± SD) | Female | Male | 1 year | 2 year | 3+ year |
| Angling (IMR) | 1978 | 43.62 ± 11.11 |  | 35.76 |  |  | 43.61 ± 11.1 |
| Angling (IMR) | 1979 | 37.92 ± 4.52 |  | 35.53 ± 0.41 |  |  | 37.91 ± 4.52 |
| Angling (IMR) | 1980 | 36.55 ± 3.25 | 35.71 ± 4.50 | 36.48 ± 3.89 |  |  | 36.55 ± 3.24 (58) |
| Angling (IMR) | 1981 | 33.63 ± 3.60 | 32.88 ± 3.77 | 33.68 ± 3.36 |  | 33.13 ± 3.28 (49) | 34.09 ± 3.84 (52) |
| Angling (IMR) | 1982 | 33.36 ± 4.28 | 34.00 ± 4.13 | 34.60 ± 4.06 | 32.10 ± 3.79 (361) | 35.96 ± 4.04 (175) |  |
| Angling (IMR) | 1983 | 30.16 ± 3.53 | 30.05 ± 3.33 | 30.38 ± 3.65 | 30.15 ± 3.52 (315) |  |  |
| Angling (NINA) | 1986 | 33.43 ± 3.27 | 33.8 ± 3.81 | 31.53 ± 1.24 |  |  | 33.43 ± 3.26 (6) |
| Angling (NINA) | 1987 | 34.71 ± 3.33 | 33.53 ± 4.48 | 35.75 ± 1.52 |  | 34.70 ± 3.33 (28) |  |
| Angling (NINA) | 1988 | 32.13 ± 3.94 | 31.36 ± 4.33 | 32.79 ± 4.43 | 32.13 ± 3.94 (89) |  |  |
| Angling (NINA) | 1989 | 29.80 | 29.80 |  | 29.8 (1) |  |  |
| Angling (NINA) | 1990 | 30.10 ± 4.59 | 29.26 ± 5.23 | 32.6 |  | 30.1 ± 4.58 (4) |  |
| Angling (NINA) | 1991 | 30.37 ± 3.66 | 28.54 ± 2.06 | 31.94 ± 4.19 | 30.36 ± 3.65 (13) |  |  |
| Angling (NINA) | 1992 | 33.27 ± 3.23 | 33.8 ± 4.38 | 32.2 |  | 33.26 ± 3.23 (3) |  |
| Angling (NINA) | 1993 | 28.43 ± 3.04 | 28.66 ± 1.74 | 28.24 ± 3.72 | 28.43 ± 3.03 (16) |  |  |
| Angling (NINA) | 1996 | 31.89 ± 4.60 | 32.98 ± 3.59 | 30.98 ± 5.67 | 31.86 ± 6.34 (14) | 31.92 ± 2.21 (15) |  |
| Angling (NINA) | 1997 | 26.50 ± 3.39 | 27.36 ± 4.72 | 25.13 ± 2.67 | 26.5 ± 3.39 (7) |  |  |
| Angling (NINA) | 1998 | 29.15 ± 3.03 | 29.7 ± 4.34 | 28.61 ± 2.10 |  | 29.15 ± 3.03 (12) |  |
| Angling (NINA) | 1999 | 29.64 ± 3.71 | 29.78 ± 3.52 | 29.52 ± 4.08 | 29.63 ± 3.71 (14) |  |  |
| Angling (NINA) | 2000 | 31.90 ± 3.64 | 31.1 ± 0.17 | 33.95 ± 7.28 |  | 31.9 ± 3.64 (6) |  |
| Angling (NINA) | 2001 | 29.89 ± 2.97 | 30.65 ± 3.05 | 29.86 ± 2.68 | 29.89 ± 2.96 (17) |  |  |
| Angling (NINA) | 2002 | 32.03 ± 4.30 | 32.68 ± 4.59 | 30.16 ± 3.77 |  | 32.03 ± 4.30 (15) |  |
| Angling (NINA) | 2003 | 29.88 ± 4.91 | 27.28 ± 3.31 | 31.74 ± 5.21 | 30.28 ± 5.68 (6) | 30.86 ± 3.34 (5) | 22.6 (1) |
| Angling (NINA) | 2004 | 35.28 ± 7.20 | 33.26 ± 6.52 | 35.33 ± 7.15 | 36.12 ± 4.32 (17) | 34.62 ± 8.85 (22) |  |
| Angling (NINA) | 2005 | 27.21 ± 5.08 | 27.04 ± 4.81 | 27.38 ± 5.70 | 28.38 ± 5.68 (16) | 27.26 ± 3.15 (11) | 25.90 ± 5.50 (15) |
| Angling (NINA) | 2006 | 27.81 ± 3.73 | 28.74 ± 2.85 | 26.3 ± 4.13 | 27.79 ± 4.06 (11) | 27.81 ± 3.68 (37) |  |
| Angling (NINA) | 2008 | 24.82 ± 3.87 | 24.26 ± 3.66 | 29.8 |  | 24.36 ± 3.81 (9) | 28.9 (1) |
| Angling (IMR) | 2009 | 31.86 ± 4.51 | 32.00 ± 4.29 | 30.48 ± 5.31 |  |  | 31.85 ± 4.50 (22) |
| Angling (NINA) | 2009 | 26.80 ± 3.13 | 27.55 ± 3.34 | 26.12 ± 2.82 |  | 28.50 ± 2.84 (9) | 26.55 ± 3.11 (62) |
| Angling (IMR) | 2010 | 26.37 ± 5.48 | 26.54 ± 5.90 | 26.62 ± 5.68 |  |  | 26.36 ± 5.48 (62) |
| Angling (NINA) | 2010 | 26.10 ± 4.30 | 26.7 ± 4.60 | 25.86 ± 4.18 | 27.6 (1) | 26.08 ± 4.31 (98) |  |
| Angling (IMR) | 2011 | 29.67 ± 5.07 | 29.96 ± 4.46 | 29.77 ± 5.84 |  | 29.60 ± 5.14 (70) | 31.19 ± 2.84 (3) |
| Angling (NINA) | 2011 | 29.00 ± 3.15 | 26.9 ± 2.54 | 29.54 ± 3.40 | 29 ± 3.14 (23) |  |  |
| Angling (IMR) | 2012 | 29.90 ± 7.23 | 26.36 ± 5.99 | 30.84 ± 7.44 | 31.20 ± 7.30 (29) |  | 26.96 ± 6.39 (13) |
| Angling (IMR) | 2013 | 31.36 ± 4.20 | 31.09 ± 4.15 | 31.22 ± 4.34 |  |  | 31.35 ± 4.20 (55) |
| Angling (IMR) | 2014 | 28.51 ± 4.07 | 28.75 ± 4.12 | 28.21 ± 3.95 |  | 28.37 ± 4.04 (260) | 28.86 ± 4.15 (99) |
| Angling (IMR) | 2015 | 25.99 ± 4.14 | 26.22 ± 4.46 | 25.84 ± 3.98 | 27.73 ± 5.07 (30) | 25.44 ± 3.87 (146) | 28.33 ± 2.66 (12) |
| Angling (IMR) | 2016 | 26.55 ± 4.38 | 25.85 ± 4.39 | 27.03 ± 4.47 | 28.68 ± 4.55 (37) | 25.59 ± 3.83 (60) | 25.68 ± 4.40 (26) |
| Angling (IMR) | 2017 | 27.74 ± 4.35 | 28.13 ± 4.91 | 27.22 ± 3.92 | 27.80 ± 4.33 (22) | 27.71 ± 4.37 (89) |  |
| Angling (IMR) | 2018 | 27.31 ± 4.58 | 27.70 ± 4.04 | 27.28 ± 4.74 | 27.31 ± 4.57 (45) |  |  |
| Trap | 2008 | 18.14 | 18.14 |  |  |  | 18.14 (1) |
| Trap | 2009 | 31.65 ± 5.01 | 31.38 ± 5.17 | 34.54 |  |  | 31.65 ± 5.01 (12) |
| Trap | 2010 | 27.90 ± 4.29 | 27.99 ± 4.58 | 27.60 ± 3.15 |  |  | 27.90 ± 4.29 (73) |
| Trap | 2011 | 28.20 ± 4.12 | 28.21 ± 3.93 | 28.19 ± 4.50 |  | 28.53 ± 4.34 (59) | 27.95 ± 3.96 (76) |
| Trap | 2012 | 27.20 ± 4.07 | 27.28 ± 3.95 | 27.15 ± 4.22 | 27.55 ± 4.92 (31) | 26.78 ± 3.93 (130) | 28.14 ± 3.76 (46) |
| Trap | 2013 | 29.52 ± 3.88 | 29.94 ± 3.88 | 29.04 ± 3.71 | 29.34 ± 3.63 (146) | 28.01 ± 3.31 (87) | 30.83 ± 4.13 (120) |
| Trap | 2014 | 28.50 ± 4.48 | 28.94 ± 4.46 | 28.06 ± 4.46 | 28.04 ± 4.23 (110) | 27.95 ± 4.14 (167) | 28.81 ± 4.63 (462) |
| Trap | 2015 | 26.13 ± 4.19 | 26.43 ± 3.94 | 25.70 ± 4.44 | 25.72 ± 3.58 (27) | 25.92 ± 4.19 (871) | 27.27 ± 4.15 (162) |
| Trap | 2016 | 27.50 ± 3.92 | 27.68 ± 3.95 | 27.27 ± 3.92 | 27.45 ± 3.76 (483) | 27.58 ± 4.06 (780) | 27.28 ± 3.69 (147) |
| Trap | 2017 | 27.67 ± 3.51 | 27.78 ± 3.85 | 27.67 ± 3.29 | 27.29 ± 3.16 (501) | 28.05 ± 3.79 (492) |  |
| Trap | 2018 | 27.84 ± 4.31 | 28.20 ± 5.68 | 27.82 ± 4.23 | 27.84 ± 4.31 (459) |  |  |

Table S2: Tukey-adjusted multiple comparisons between the decades of the generalised linear model for the angled fish. The top diagonal represents the difference in average marine growth (cm) between two smolt year classes. The bottom diagonal represents the Tukey-adjusted p values resulting from the multiple comparisons. Significant comparisons are shown in bold. SE; standard error, df; degrees of freedom, MG difference; difference in average marine growth (cm) for each comparison.

| MG difference (cm) | contrast | estimate | SE | df | t.ratio | p.value |
| --- | --- | --- | --- | --- | --- | --- |
| 2.72 | 80s - 90s | 1.09 | 0.02 | 2041 | 6.15 | <0.000 |
| 3.71 | 80s - 00s | 1.17 | 0.01 | 2041 | 12.97 | <0.000 |
| 5.00 | 80s - 10s | 1.22 | 0.01 | 2041 | 25.92 | <0.000 |
| 0.99 | 90s - 00s | 1.07 | 0.02 | 2041 | 3.70 | 0.001 |
| 2.28 | 90s - 10s | 1.12 | 0.02 | 2041 | 7.17 | <0.000 |
| 1.29 | 00s - 10s | 1.04 | 0.01 | 2041 | 3.65 | 0.002 |
|  |  |  |  |  |  |  |
|  |  |  |  |  |  |  |
|  |  |  |  |  |  |  |
|  |  |  |  |  |  |  |
|  |  |  |  |  |  |  |
|  |  |  |  |  |  |  |
|  |  |  |  |  |  |  |

Table S3 Tukey-adjusted multiple comparisons between the sea ages within smolt year classes (2012 – 2016) of the generalised linear model for the trap fish. Significant comparisons are shown in bold. SE; standard error, df; degrees of freedom.

|  | contrast | ratio | SE | df | t.ratio | p.value |
| --- | --- | --- | --- | --- | --- | --- |
| 2012 | **1SW - 2SW** | 1.02 | 0.03 | 3511 | 0.60 | 0.821 |
|  | **1SW - 3+SW** | 0.96 | 0.03 | 3511 | -1.35 | 0.368 |
|  | **2SW - 3+SW** | **0.94** | **0.02** | **3511** | **-2.71** | **0.018** |
| 2013 | **1SW - 2SW** | **1.06** | **0.02** | **3511** | **3.18** | **0.004** |
|  | **1SW - 3+SW** | 0.96 | 0.02 | 3511 | -2.08 | 0.093 |
|  | **2SW - 3+SW** | **0.91** | **0.02** | **3511** | **-5.58** | **<0.000** |
| 2014 | **1SW - 2SW** | 1.01 | 0.02 | 3511 | 0.37 | 0.929 |
|  | **1SW - 3+SW** | 0.97 | 0.02 | 3511 | -1.72 | 0.199 |
|  | **2SW - 3+SW** | **0.96** | **0.01** | **3511** | **-2.74** | **0.017** |
| 2015 | **1SW - 2SW** | 1.00 | 0.03 | 3511 | 0.00 | 1.000 |
|  | **1SW - 3+SW** | 0.95 | 0.03 | 3511 | -1.52 | 0.283 |
|  | **2SW - 3+SW** | **0.95** | **0.01** | **3511** | **-3.88** | **0.000** |
| 2016 | **1SW - 2SW** | 1.00 | 0.01 | 3511 | 0.16 | 0.986 |
|  | **1SW - 3+SW** | 1.01 | 0.01 | 3511 | 0.51 | 0.864 |
|  | **2SW - 3+SW** | 1.01 | 0.01 | 3511 | 0.45 | 0.895 |
